# Mycotoxin Adducts on Human Serum Albumin: Biomarkers of Exposure to *Stachybotrys chartarum*

**DOI:** 10.1289/ehp.9064

**Published:** 2006-04-26

**Authors:** Iwona Yike, Anne M. Distler, Assem G. Ziady, Dorr G. Dearborn

**Affiliations:** 1 Departments of Pediatrics and; 2 Pharmacology and; 3 Mary Ann Swetland Center for Environmental Health, Case Western Reserve University, Cleveland, Ohio, USA

**Keywords:** biomarkers, satratoxin G, *Stachybotrys chartarum*, trichothecenes

## Abstract

**Objective:**

Despite the growing body of evidence showing adverse health effects from inhalation exposure to the trichothecene-producing mold *Stachybotrys chartarum*, controversy remains. Currently, there are no reliable assays suitable for clinical diagnosis of exposure. We hypothesized that satratoxin G (SG)–albumin adducts may serve as biomarkers of exposure to this fungus.

**Design:**

We studied the formation of adducts of SG with serum albumin *in vitro* using Western blots and mass spectrometry (MS) and searched for similar adducts formed *in vivo* using human and animal serum.

**Results:**

Samples of purified human serum albumin that had been incubated with increasing concentrations of SG showed concentration-dependent albumin bands in Western blots developed with anti-SG antibodies. MS analysis found that as many as 10 toxin molecules can be bound *in vitro* to one albumin molecule. The sequencing of albumin-adduct tryptic peptides and the analysis of pronase/aminopeptidase digests demonstrated that lysyl, cysteinyl, and histidyl residues are involved in the formation of these adducts. Serum samples from three patients with documented exposure to *S. chartarum* similarly revealed lysine–, cysteine–, and histidine–SG adducts after exhaustive digestion, affinity column enrichment, and MS analysis. These adducts were also found in the sera from rats exposed to the spores of *S. chartarum* in contrast to control human subjects and control animals.

**Conclusions:**

These data document the occurrence of SG–albumin adducts in both *in vitro* experiments and *in vivo* human and animal exposures to *S. chartarum*.

**Relevance to clinical practice:**

SG–amino acid adducts may serve as reliable dosimeter biomarkers for detection of exposure to *S. chartarum*.

The filamentous mold *Stachybotrys chartarum* requires water-saturated cellulose to grow, and when found in an indoor environment, it is an indicator of significant water intrusion and damage. Although *S. chartarum* produces several classes of mycotoxins, of greatest concern are macrocyclic trichothecenes, the most potent members of a large family of tri-chothecenes (Miller et al. 2003). These mycotoxins bind to a single site on eukaryotic ribosomes and directly inhibit initiation, elongation, or termination of protein synthesis depending on which trichothecene is bound (Feinberg and MacLaughlin 1989). There are two chemotypes of *S. chartarum*, one producing several macrocyclic trichothecenes and another that produces atranones and simple trichothecenes (e.g., trichodermin) but never any of the macrocyclic trichothecenes (Andersen et al. 2002). Although animal models of pulmonary injury demonstrate that macrocyclic trichothecenes are not the only source of lung damage, tracheal instillation of spores containing these mycotoxins results in significantly more acute injury (Leino et al. 2003; Yike and Dearborn 2004; Yike et al. 2005).

Concern about *S. chartarum* in indoor environments surfaced in the mid-1980s (Croft et al. 1986). Case reports of exposures in both residential and the nonindustrial work-place suggested that chronic indoor exposures could result in a variety of debilitating respiratory and nonrespiratory symptoms, perhaps including an effect on immune function (Hodgson et al. 1998; Johanning et al. 1996). Recent reviews on toxic mold–related health effects raise both concern and controversy (Horner 2005; Hossain et al. 2004).

In 1994 we recognized an outbreak of acute pulmonary hemorrhage in young infants in Cleveland (Montana et al. 1997). The initial case–control investigation led by the Centers for Disease Control and Prevention (CDC) found an association of this often fatal disorder with the presence of *S. chartarum* in the water-damaged homes of these infants (Etzel et al. 1998). Although subsequent review by the CDC of the initial field investigation of 10 infant cases and 30 controls has questioned the strength of the association (Etzel 2003), we have subsequently cared for an additional 30 cases with the continued observation that almost 90% of these infants come from homes with documented *S. chartarum* (Dearborn et al. 1999, 2002). Others have also seen pulmonary hemorrhage in infants with toxigenic mold exposure (Miller et al. 2003), and informal surveillance identified more than 100 other cases of acute infant pulmonary hemorrhage diagnosed across the United States between 1993 and 1997 (Dearborn et al. 1999). The non-pulmonary manifestations are similar to those described in animals exposed to *S. chartarum* and are consistent with the immune suppressive, neurotoxic, and hemolytic effects of the trichothecenes and/or accompanying mycotoxins (Dearborn et al. 2002).

Conclusive evidence regarding the etiology of the infant pulmonary hemorrhage disorder and other adverse health effects linked to exposure to this mold awaits the development of proper biomarkers. At present, there is no reliable way to determine human exposure to *S. chartarum* (Dillon et al. 1999; Miller et al. 2003). Finding this mold in a patient’s home or work environment remains circumstantial evidence without a biological marker documenting the extent and timing of the exposure. Whether it is infants, older children, or adults, it is very apparent that the controversy surrounding the significance of inhalation exposure to “toxic mold” will continue until quantitative, dosimeter biomarkers are available and used in proper epidemiologic studies.

In this report, we present evidence that satratoxin G (SG), a macrocyclic trichothecene produced by *S. chartarum* (Figure 1), forms covalent adducts with serum albumin. These adducts may serve as biomarkers of exposure to *S. chartarum* similar to the serum albumin adducts that have been used as exposure dosimeters for another mycotoxin, aflatoxin B_1_, and other xenobiotics and chemicals (Groopman and Kensler 1999).

## Materials and Methods

### Experimental design

Figure 2 is a summary of the *in vitro* and *in vivo* experiments and analyses described in this article.

### Animal experiments

#### Animals

Sprague-Dawley rats were obtained from Charles River (Wilmington, MA). All animals were housed in microisolators in the Case Western Reserve University animal facility and fed the standard diet of Teklad 8664 (Harlan, Madison, WI) and water *ad libitum*. The animal research protocol has been approved by the Case Western Reserve University Institutional Animal Care and Use Committee. The animals were treated humanely and with regard for the alleviation of suffering.

#### Intratracheal instillation of fungal spores

Seven-day-old rat pups weighing around 10 g were used for measurements of SG in blood. Male and female rats weighing 100 g were used to search for SG–albumin adducts. In both cases, the animals were anesthetized with isoflurane (Baxter, Deerfield, IL). A transverse skin incision was made, and the trachea was exposed by blunt dissection. *S. chartarum* spores (isolate JS58-17; 4.0 × 10^5^ spores per gram body weight in SG measuring experiments and 0.5 × 10^5^ spores/gram body weight in albumin adduct detection experiments) suspended in 50 μL of phosphate-buffered saline (PBS) containing 0.1% Tween-20 were injected directly into the trachea using a 24 G angiocatheter attached to a sterile Hamilton syringe. The incision was closed and treated with New Skin liquid bandage (Medtech Laboratories Inc., Jackson, WY) to facilitate healing.

#### Collection of blood

The animals were exsanguinated through the right ventricle under isoflurane anesthesia at indicated times after spore instillation, and the blood samples were combined using three animals per time point. For SG measurements, the blood was immediately extracted with ethanol (see below), whereas for albumin adduct detection, the blood was centrifuged at 1,000 × *g* for 10 min and the serum aspirated and stored at −20°C.

### Human blood samples

Blood samples were obtained from three adult patients with documented exposure to *S. chartarum* who were evaluated at the Environmental Health Clinic, Swetland Center for Environmental Health, Case Western Reserve University (Cleveland, OH). The use of human samples was reviewed and approved by the Institutional Review Board of the University Hospitals of Cleveland, and all recruited patients gave informed consent. The sample from patient 1 was taken approximately 2 months after the completion of mold remediation in her home. Previous air sampling in her living room had measured 4,700 *S. chartarum* spores/m^3^ (Air-O-Cell; US Micro-Solutions Inc, Greensburg, PA). Blood samples from patients 2 and 3 were obtained while they were still living in their home where *S. chartarum* was found in their bedroom carpeting (1,030 *S. chartarum* spore equivalents/g; quantitative polymerase chain reaction, P&K Microbiology Services, Cherry Hill, NJ). Control blood samples were received from the investigators. The blood was centrifuged and the serum stored at −20°C.

### Anti-SG antibodies and SG ELISA

Polyclonal antibodies against SG were generously donated by J. Pestka, Michigan State University (East Lansing, MI). The IgG fraction, obtained by ammonium sulfate precipitation from the sera of rabbits immunized with the conjugates of bovine serum albumin and SG (Chung et al. 2003), was purified by passing through an affinity column of bovine serum albumin (Sigma-Aldrich, St. Louis, MO) conjugated to Amino Link Sepharose (Pierce, Rockford, IL) to remove antibodies specific for bovine albumin epitopes.

SG was measured by ELISA (detection limit = 0.1 ng/mL) in 1:1 whole blood ethanol extracts according to the method of Chung et al. (2003) using anti-SG antibody and horseradish peroxidase–SG conjugates provided by J. Pestka.

### *Formation of albumin–SG adducts* in vitro

Human serum albumin (HSA) isolated from human blood (Sigma-Aldrich) and/or recombinant HSA (rHSA; GTC Biotherapeutics, Spencer, MA) was dissolved in PBS at 10 μM concentration and incubated for 20 hr at 37°C with 0-, 1-, 2-, 5-, 10-, and 20-molar excess of SG (gift from B. Jarvis, University of Maryland). The samples were then subjected to SDS–PAGE followed by Western blots or dialyzed and analyzed by mass spectrometry (MS) as described below. A molar ratio of 1:10 of albumin to SG was used for MS studies.

*N*-α-Acetyl-l-lysine (Sigma-Aldrich) was dissolved in PBS at 10 μM concentration and incubated for 20 hr at 37°C with an equimolar concentration of SG.

### Electrophoresis and Western blot analysis

SDS–PAGE was performed using 12% precast Criterion gels (Bio-Rad, Hercules, CA). The proteins were transferred to nitrocellulose, and Western blots were developed using 1:1,000-diluted (affinity purified as above) anti-SG antibody. Alkaline phosphatase anti-rabbit IgG (Sigma-Aldrich) was used as a secondary antibody, and the protein bands were visualized with an alkaline phosphatase substrate kit (Bio-Rad).

### Preparation of samples for MS analysis

#### Tryptic digestion of rHSA and human serum

rHSA was incubated with (1:10 molar ratio of rHSA to SG) and without SG as described above and dialyzed against 20 mM ammonium bicarbonate, pH 7.8. Dialyzed protein was incubated overnight at 37°C with sequencing-grade trypsin (Promega, Madison, WI) at 1:100 wt/wt ratio. Samples of human serum (1–3 mL) were similarly digested with a TCPK trypsin preparation (Sigma-Aldrich).

#### Exhaustive digestion of rHSA and serum

rHSA and rHSA–SG adducts, dialyzed overnight against 20 mM ammonium bicarbonate pH 7.8, were incubated overnight at 37°C with pronase (Calbiochem, EMD Biosciences, San Diego, CA) at 1:100 wt/wt ratio and leucine aminopeptidase (Sigma-Aldrich) at 1:1,000 wt/wt ratio. Human and rat serum (1–3 mL) were similarly digested except using the leucine aminopeptidase at 1:500 wt/wt. If the digestion to single amino acids was not complete, carboxypeptidase Y (Sigma-Aldrich) was added to reconstituted samples for 4 hr and the analysis was repeated.

#### Affinity chromatography

Both trypsin and pronase digested samples were heat inactivated at 60°C for 30 min and centrifuged at 17,000 × *g* for 20 min before affinity chromatography. The affinity column for isolating peptide and amino acid adducts was prepared by conjugating anti-SG antibody (see above) to AminoLink Sepharose (Pierce, Rockford, IL). Heat-inactivated tryptic and/or pronase digests from human and rat serum were loaded onto the column and incubated for 1 hr at room temperature. The column was extensively washed with PBS followed by 20 mM ammonium bicarbonate, pH 7.8. Bound adducts were eluted with 0.02% formic acid and evaporated. The samples were reconstituted in 0.1% formic acid and analyzed by MS.

### MS instrumentation and analyses

#### Intact protein

Intact rHSA was analyzed using an Applied Biosystems (Framingham, MA) Q-STAR XL quadrupole time-of-flight (TOF) mass spectrometer equipped with a nanospray source or a Bruker (Billerica, MA) Biflex III TOF mass spectrometer with a matrix-assisted laser desorption ionization (MALDI) source. For intact protein analysis by MALDI–TOF–MS, sinapinic acid was used as the matrix. One microliter of the saturated matrix mixture (in a 1:1 acetonitrile: water solution) was spotted on target with 1 μL of the protein solution.

#### Tryptic peptide identification

After rHSA digestion with trypsin, the resulting peptides were analyzed using MALDI–TOF–MS to determine their molecular weights. For MALDI–TOF–MS analysis of the peptides, the matrix α-cyano-4-hydroxy cinnamic acid was used. One microliter of the saturated matrix (in a 1:1 acetonitrile:water solutions) was spotted on target with 1 μL of the analyte. Using the *m*/*z* values from the mass spectra, peptide mass fingerprinting (PMF) was performed to determine the identity of the peptides by matching the experimental molecular weights with the theoretical values calculated from the protein sequence.

To further confirm the identity of the peptides and locate the modified amino acids, tandem MS (MS–MS) was performed using the ThermoElectron LCQ-Deca XP (Thermo-Electron Corp., Waltham, MA) plus ion trap mass spectrometer with nanospray source. Tryptic digests were diluted in 1% acetic acid, and 2 μL of each sample were pressure injected onto a self-packed 10 cm × 75 μm inner-diameter Phenomenex Jupiter (Phenomenex Inc., Torrance, CA) C_18_ reversed-phase capillary column. The peptides were eluted from the column by an acetonitrile and aqueous 0.05 M acetic acid gradient with a flow rate of approximately 0.25 μL/min at the nanospray tip. The digest was analyzed by acquiring full scan mass spectra followed by MS–MS. The three most abundant ions detected in the full scan mass spectrum were then selected and fragmented to yield the MS–MS spectrum of the peptide. The MS–MS data were analyzed using the ThermoElectron BioWorks 3.1 program (ThermoElectron). All matching spectra were verified manually.

The pronase digests were analyzed on the MALDI–TOF mass spectrometer using the same matrix and sample preparation detailed above for the tryptic digest analysis. The limit of detection for this analysis of human samples was approximately 10 nmol/mL of serum.

## Results

### Detection of SG in blood

When 7-day-old infant rat pups were exposed intratracheally to high doses (4.0 × 10^5^ spores/gm body weight) of highly toxic *S. chartarum* (isolate JS58-17), SG was detected in ethanol extracts of the whole blood by SG ELISA only immediately after instillation (Figure 3). The level of free toxin decreased below the detection limit within the next 15 min. No immunoreactive free toxin could be detected in the sera of exposed animals between 1 and 72 hr postexposure.

### *SG adducts* in vitro

#### Detection of anti-SG–reactive albumin

Samples of purified HSA (Sigma-Aldrich) were incubated with increasing concentrations of SG (PBS, 37°C, 20 hr) and subjected to SDS–PAGE after reduction and boiling of the samples. Western blots using anti-SG antibody clearly demonstrate the HSA band at approximately 67 kDa (Figure 4) with the intensity of staining increasing with increasing concentrations of SG. This concentration dependence of the SG staining that persists through boiling and SDS electrophoresis supports the formation of covalent SG–albumin adducts. These results were confirmed using rHSA.

#### MS analysis of intact protein

When untreated and SG-incubated rHSA (20 hr, 37°C, PBS, 1:10 protein to toxin ratio) were analyzed using an electrospray ionization (ESI) ESI-quadrupole TOF mass spectrometer, a mass shift was seen in the treated rHSA sample. This molecular weight increase was indicative of as many as 10 satratoxin molecules bound to the protein. These results were further confirmed using a MALDI–TOF mass spectrometer (data not shown).

#### MS analysis of SG–*N*-acetyl-l-lysine adduct

Because the ɛ-amines of lysyl residues are a likely site of SG nucleophilic attack, we incubated the toxin with *N*-α-acetyl-l-lysine (1:1 molar ratio, 37°C, 20 hr) and analyzed the resulting SG–lysyl adduct. Figure 5A and B, shows the spectra from both MALDI–TOF and ESI MS–MS of the resulting SG–lysyl adduct. The *m*/*z* 716 is consistent with the addition of 528 Da, an apparent loss of oxygen when the SG bound to the amino acid. When the ions at *m*/*z* 716 were isolated and fragmented, the MS/MS spectrum contained a peak at *m*/*z* 172 representing the *N*-acetyl lysine as well as a peak representing a fragment of the toxin at *m*/*z* 239, a convenient marker for this adduct in MS–MS experiments. This peak at 239 Da is detected both in the mass spectrum of the toxin molecule alone and as a fragment ion in the MS–MS spectrum of the toxin molecule.

#### Detection of adducted tryptic peptides from rHSA

To locate the sites of SG adduction to rHSA, both untreated and treated rHSA were digested with trypsin and analyzed with MALDI–TOF MS using PMF to identify the peptides. When analyzing the tryptic peptides of treated rHSA, several ions were detected that were consistent with the predicted mass of peptides bound to one SG molecule (Table 1). To identify the exact position of toxin molecules on these peptides, the tryptic digests of treated and control rHSA were analyzed further using ESI MS–MS (Table 1, Figure 6). Around 60% coverage was obtained for both samples. The ions representing tryptic peptides were in the triply charged state. When the ions at *m*/*z* 528 were isolated and fragmented, the resulting MS–MS spectrum identified the peptide as K(SG528)YLYEIAR with a calculated mass of 1,582 [(528 × 3) − 2]. Another labeled peptide was identified when the ions at *m*/*z* 475 were isolated and fragmented. The fragmentation pattern in this MS–MS spectrum is consistent with the peptide LC(SG544)TVATLR with a calculated mass of 1,423 Da [(475 × 3) − 2]. Last, when the ions at *m*/*z* 469 were fragmented, the same peptide was identified, but with a +528 SG adduct [(469 × 3) − 2] = 1,405 Da. As indicated in Table 1, the sequences of adducted tryptic peptides all contained lysyl, cysteinyl, or histidyl residues (see below), residues that are likely to be susceptible to modification by the toxin epoxide groups.

#### Detection of adducted amino acids in pronase digests of rHSA

To further characterize the rHSA–SG reactivity, exhaustive proteolysis of reacted rHSA was performed with pronase and leucine aminopeptidase. The modified amino acids were purified using anti-SG antibody affinity chromatography and analyzed by MALDI–TOF–MS. In the MALDI–TOF mass spectra, several ions were detected corresponding to the amino acids lysine (Lys), histidine (His), and cysteine (Cys), each containing one SG molecule (Figure 7A). Two different cysteinyl adducts of +528 and +544 Da were detected. The amino acid assignments in Figure 7A were confirmed by ESI MS–MS (data not shown).

### *SG–albumin adducts* in vivo

#### Amino acid SG adducts in pronase digests of human and rat sera

Subsequently, 2 mL serum samples from three patients with documented exposure to *S. chartarum* and three control subjects were digested with pronase and leucine aminopeptidase, and the adducts were affinity purified and analyzed by MALDI–TOF MS. In Figure 7B, the three top spectra in each panel were acquired from the samples from exposed patients, whereas the bottom spectra are from one of the control subjects. Patients 2 and 3 had a more recent exposure compared to patient 1. Although cysteinyl adducts were detected in all three of the exposed patient samples, lysyl and histidyl adducts were not detected in patient 1, whose blood sample was collected 2 months after the termination of exposure. No amino acid adducts were detected in the sera from the three control subjects. Similarly, cysteine–, lysine–, and histidine–SG adducts were detected after exhaustive proteolysis of the sera of rats exposed intratracheally to *S. chartarum* collected 6 hr after the instillation of fungal spores (Figure 8). No adducts could be found in parallel samples from control animals.

#### Adducted tryptic peptides from serum of a patient exposed to *S. chartarum.*

To demonstrate the albumin origin of amino acid adducts detected in samples from human subjects, serum from patient 3 (most recent exposure, all three amino acyl adducts detected) was digested with trypsin and the adducted peptides isolated with anti-SG immunoaffinity chromatography. Analysis by MALDI–TOF–MS followed with PMF detected eight peptides with the additional mass of 528 Da and four with 544 Da (Table 2). All of the detected tryptic peptides contained at least one of the three amino acyl residues, lysyl, cysteinyl, and histidyl, that were identified as probable modification sites in rHSA.

## Discussion

In this study we have demonstrated that SG, a macrocylic trichothecene produced by *S. chartarum*, forms covalent adducts with HSA and that these adducts can be detected in clinical samples from patients exposed to this fungus.

**Figure 1:**
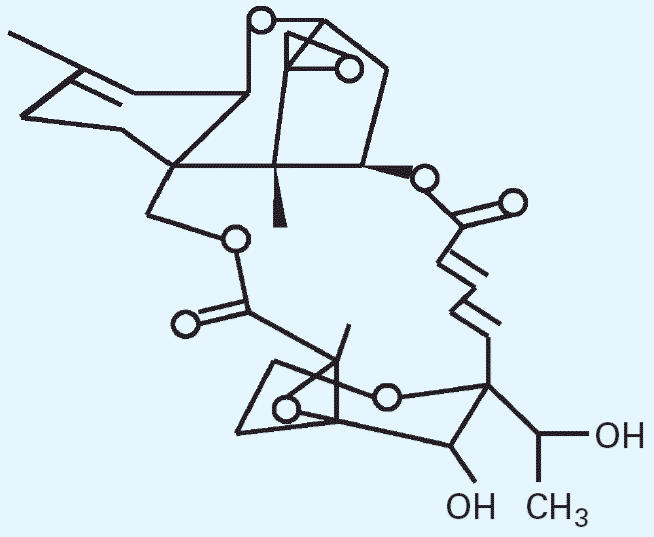
Structure of SG. Abbreviations: OH, hydroxyl group; CH_3_ , methyl group.

**Figure 2:**
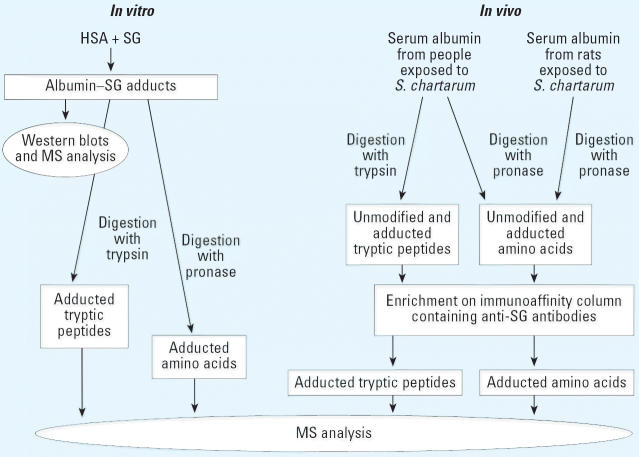
Experimental design.

Serum albumin adducts have been used as exposure dosimeters for other toxic agents including another mycotoxin, aflatoxin B_1_ (Groopman and Kensler 1999; Guengerich et al. 2002; Sabbioni et al. 1990). This potent carcinogen causing hepatocellular carcinoma is oxidized in the liver by cytochrome P450 to the epoxide that then reacts with the ɛ-amine of lysyl residues either directly or through the spontaneously formed dialdehyde. The five primary macrocyclic trichothecenes produced by *S. chartarum* are SG (Figure 1), satratoxin H, isosatratoxin F, roridin E, and verrucarin J. All contain an epoxide group that is critical to their toxicity, and the first and third of this series contain a second epoxide (Andersen et al. 2002; Jarvis et al. 1998). These highly reactive groups are likely to be involved in rapid adduct formation with proteins, and with inhalation exposure, this reaction would likely occur with blood proteins in the alveolar capillaries.

**Figure 3:**
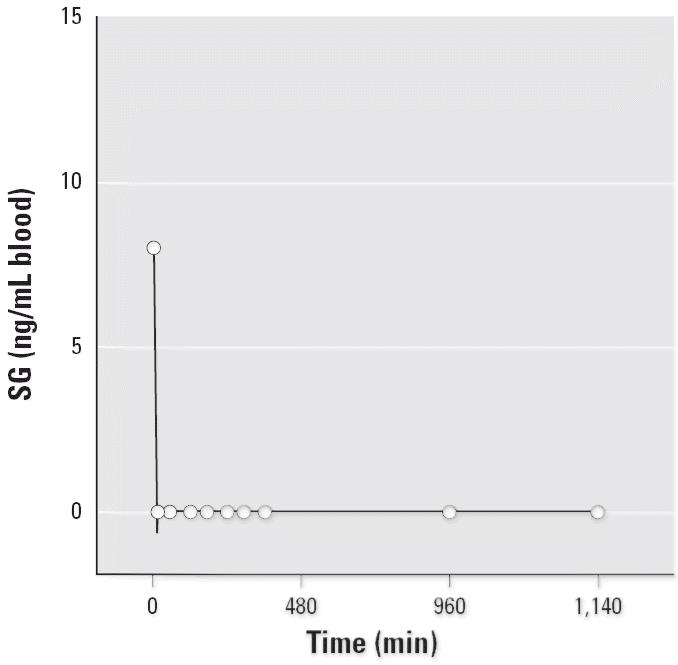
SG levels in the blood of infant rats exposed to the spores of *S. chartarum*.

The formation of mycotoxins adducts is consistent with our observation that free SG could be detected in the blood of rat pups exposed to the spores of *S. chartarum* only immediately after exposure (Figure 3), and with previous observations of rapid removal and/or metabolism of trichothecenes (Swanson and Corley 1989). Reaction with serum albumin and other blood proteins is likely responsible for the rapid disappearance of free toxin from the blood.

**Figure 4:**
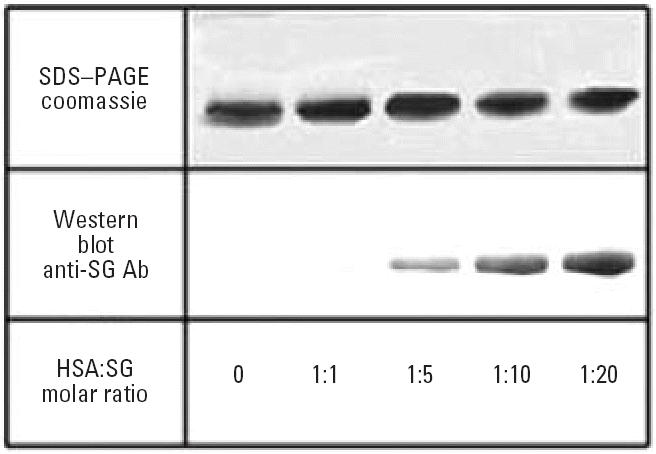
SDS–PAGE and Western blots of HSA incubated with different concentrations of SG (3 μg protein/lane). Western blots were developed with affinity-purified anti-SG antibody (Chung et al. 2003).

**Figure 5:**
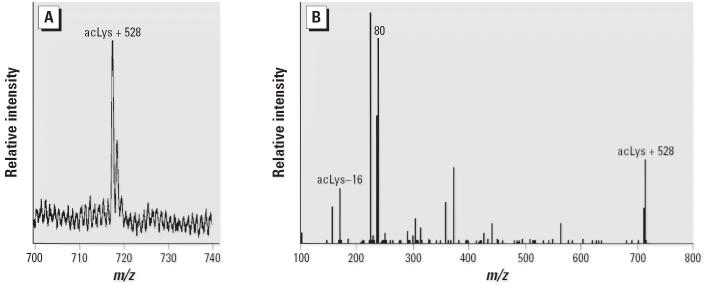
(*A*) Portion of the MALDI–TOF mass spectrum of *N*-α-acetyl-l-lysine (acLys) incubated with equimolar concentration of SG. A peak is detected at *m*/*z* 716.8. (*B*) ESI MS–MS spectrum of the ions at *m*/*z* 716.8.

**Figure 6:**
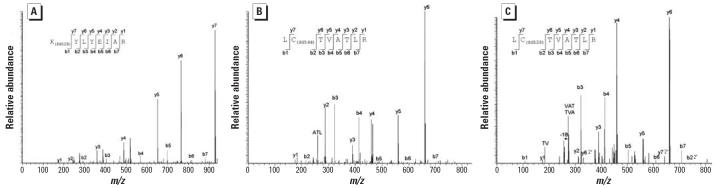
ESI MS–MS of tryptic peptides from rHSA containing SG modifications. (*A*) Ions at *m*/*z* 528.33. (*B*) Ions at *m*/*z* 474.64. (*C*) Ions at *m*/*z* 469.17. All spectra shown are for tryptic peptides in the triply charged state. In the spectra, the fragment ions have been labeled according to Biemann nomenclature (Biemann 1990). To form b and y ions, the peptide is fragmented at the amide bonds along the peptide backbone with the b ions containing the N-terminus and the y ions containing the C-terminus. The sequences of the peptides are shown in the figure with the fragmentation of the peptide backbone indicated.

**Figure 7:**
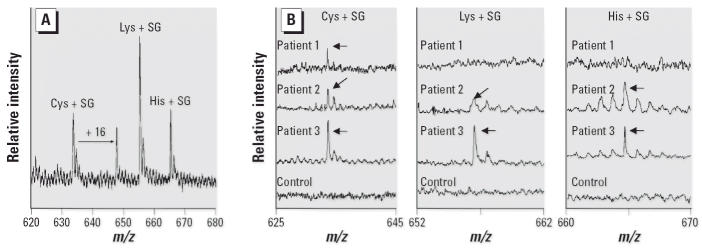
MALDI–TOF spectra of amino acid–SG adducts in pronase digests of rHSA (*A*) and amino acid–SG adducts in pronase digests of HSA (*B*) from *S. chartarum*–exposed and control patients.

**Figure 8:**
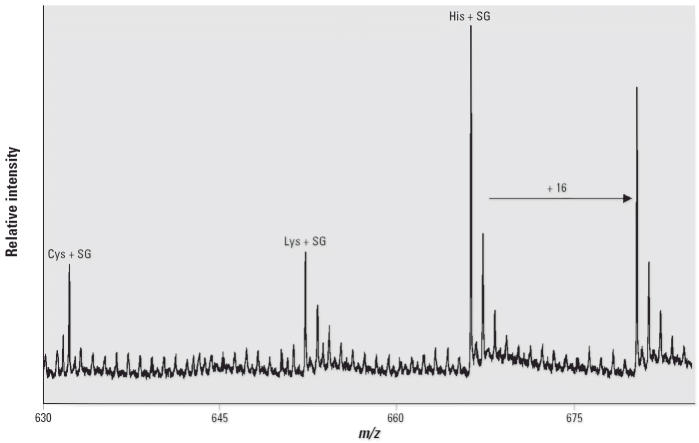
MALDI–TOF spectra of cysteine–, lysine–, and histidine–SG adducts from serum of rats exposed to the spores of *S. chartarum*.

The reaction of SG with tissue and cellular proteins is also likely. Our first experimental data suggesting the formation of SG–protein adducts was the detection of immunoreactive satratoxin in murine tissue sections and cells obtained by bronchoalveolar lavage. The presence of satratoxin epitopes in lung sections that had been extensively rinsed in organic solvents during fixation and staining (Gregory et al. 2004) indicated that the toxin might be covalently bound to tissue/cell components. Western blots showing the staining of albumin with anti-SG antibody (Figure 4) were obtained with reduced and boiled samples, further suggesting covalent links between the mycotoxin and protein.

ESI TOF and MALDI–TOF–MS analysis of the intact adducted protein showed that up to 10 amino acid residues in the albumin molecule are modified after the incubation of the protein with a 20-fold excess of toxin *in vitro*. The extent of albumin modification *in vivo* would be dependent upon the level and timing of exposure and is likely to reflect the cumulative nature of chronic exposure. MALDI–TOF–MS analysis of exhaustive pronase/aminopeptidase digests of rHSA demonstrated that in addition to lysyl residues, two other amino acyl residues, cysteinyl and histidyl, are involved in adduct formation. All of the rHSA-derived tryptic peptides with SG adducts had one of those amino acyl residues within their sequences, and Cys75 and Lys137 were positively identified as modification sites using ESI MS–MS analysis.

Using an affinity column with anti-SG antibodies to isolate adducts from proteolytic digests provides a concentrating step that greatly increases the sensitivity of detection. Our ability to detect Lys–, Cys–, and His–SG adducts in pronase/aminopeptidase digests of serum from patients exposed to *S. chartarum* in their homes, in contrast to samples from people without mold exposure, demonstrates the feasibility of a practical biomarker assay. Detection of modified tryptic peptides with albumin sequences in the sample of patient’s serum indicates that those amino acyl adducts came from serum albumin, although modification of other serum proteins is likely. In addition, the presence of Lys–, Cys–, and His–SG adducts in the sera from rats exposed intratracheally to the spores of a highly toxic isolate of *S. chartarum* further confirms the biomarker potential of the adducts.

An SG ELISA [the same assay developed by Chung et al. (2003) used in this study to measure SG in blood, as described in “Materials and Methods”] was used by Brasel et al. (2004) to detect the toxin in organic solvent extracts of the sera of patients exposed to molds through indoor air inhalation. Their removal of proteins before analysis precludes the detection of albumin–toxin adducts, although some amino acid and small peptide toxin adducts may be present in those extracts. Most of their positive clinical samples contained immunoreactive toxin very close to the limit of detection. MS analysis detected possible toxin breakdown products with spectral properties similar to those of macrocyclic trichothecenes, but the exact nature and origin of the detected substance are not known. For this approach to be quantitatively useful in assessing *S. chartarum* exposure, the nature and kinetics of SG metabolism in humans must be better understood. In addition, our animal experiments showing rapid loss of detectable free toxin from the blood after inhalation-type exposure suggest this analytical approach to be of limited value.

Other methods to document *S. chartarum* exposure include reverse-transcriptase polymerase chain reaction (RT-PCR) using species-specific genomic probes and an ELISA for a hemolysin produced by this fungus. Although quantitative PCR is quite sensitive (Haugland et al. 1999), the collection of proper secretion samples often requires invasive procedures (e.g., bronchoscopy) in order to collect secretions likely to contain the fungus, which is also a time-limited sampling opportunity. In addition, detection using RT-PCR does not discriminate between the isolates that produce macrocyclic trichothecenes and those that do not. Similarly, the use of the hemolysin as a biomarker does not distinguish between macrocyclic trichothecene producers and non-producers because all of the tested isolates of *S. chartarum* seem to produce stachylysin (Vesper et al. 1999, 2001). The polyclonal antibodies currently used in the Stachylysin–ELISA assay (Van Emon et al. 2003) recognize fungal antigens from other species, such as *Penicillium chrysogenum* (Yike I, unpublished data). A more specific, monoclonal antibody may be needed in order to develop a reliable assay. A monoclonal antibody developed recently against a spore-specific antigen from *S. chartarum* recognizes a secreted protein found both in highly toxic isolates and in those with low toxicity. However, because of its relatively low abundance, this protein may be difficult to detect in human samples (Schmechel et al. 2006).

Although the exact structures and mechanisms of the SG adduct formation require further studies, our ability to detect these adducts in blood samples of individuals and animals exposed to *S. chartarum* in contrast to control subjects indicates a high potential for this approach to provide a practical, quantitative dosimeter. Serum albumin is one of the most abundant proteins (~ 60 mg/mL) with a half-life of approximately 20 days. This makes it a good candidate for a dosimeter of both acute (days) and fairly recent (weeks to several months) mold exposure. Our results suggest that SG–albumin adducts may serve as quantitative biomarkers of inhalation exposure to *S. chartarum* and other satratoxin-producing fungi because they can be readily detected in small samples of blood.

## Figures and Tables

**Table 1:** Adducted tryptic peptides from rHSA.

*m*/*z*	Amino acids	Sequence^a^	Adduct
5739.9	277–323	E**CC**E**K**PLLE**K**S**HC**IAEVENDEMPADLPSLAADFVES**K**DV**CK**NYAEAK	528
4566.2	214–276	V**H**TE**CCH**GDLLE**C**ADDRADLA**K**YI**C**ENQDSISS**K**LK	544
4565.2	485–519	RP**C**FSALEVDETYVP**K**EFNAETFTF**H**ADI**C**TLSEK	528
3487.5	82–106	ETYGEMAD**CC**A**K**QEPERNE**C**FLQ**H**K	528
2551.5	223–240	FP**K**AEFAEVS**K**LVTDLTK	528
2551.5	467–484	TPVSDRVT**KCC**TESLVNR	544
1914.5	263–274	YI**C**ENQDSISSK	528
1582.6	137–144	**K**YLYEIAR	528
2583.3	145–160	R**H**PYFYAPELLFFA**K**R	528
2743.4	175–195	AA**C**LLP**K**LDELRDEG**K**ASSAK	528
2264.7	219–233	LSQRFP**K**AEFAEVSK	528
1995.5	337–348	R**H**PDYSVVLLLR	528
1719.2	277–286	E**CC**E**K**PLLEK	528
1404.5	74–81	L**C**TVATLR	528
1420.5		L**C**TVATLR	544
1353.6	535–541	**HK**P**K**ATK	544
1014.2	1–4	DA**H**K	544
1037.4	535–538	**HK**PK	528
2599.3	145–160	R**H**PYFYAPELLFFA**K**R	544

a Presumed modification sites of peptides analyzed by MALDI–TOF–MS are in bold. Modification sites identified by ESI MS–MS are underlined.

**Table 2:** Tryptic peptides from serum albumin of a patient exposed to *S. chartarum*.

*m*/*z*	Amino acids	Sequence^a^	Adduct
1067.4	435–438	YT**K**K	528
1109.4	464–468	**H**PEAK	528
1226.2	29–34	SEVA**H**R	528
1275.2	215–221	ASSA**K**QR	528
1418.4	222–229	L**KC**ASLQK	528
1544.9	89–97	SL**H**TLFGDK	528
1720.3	301–310	E**CC**E**K**PLLEK	528
2911.1	76–97	T**C**VADESAEN**C**D**K**SL**H**TLFGDK	528
1053.4	559–562	**HK**PK	544
1564.0	234–242	AF**K**AWAVAR	544
2035.2	550–562	QTALVELV**K**H**K**PK	544
3910.1	37–65	DLGEENF**K**ALVLIAFAQYLQQ**C**PEED**H**VK	544

a Amino acid residues that may be modified within the peptide are in bold.
